# The relation between cartilage biomarkers (C2C, C1,2C, CS846, and CPII) and the long-term outcome of rheumatoid arthritis patients within the CAMERA trial

**DOI:** 10.1186/ar3331

**Published:** 2011-05-08

**Authors:** Marije F Bakker, Suzanne MM Verstappen, Paco MJ Welsing, Johannes WG Jacobs, Zalima N Jahangier, Maaike J van der Veen, Johannes WJ Bijlsma, Floris PJG Lafeber

**Affiliations:** 1 Department of Rheumatology & Clinical Immunology, University Medical Center Utrecht, P.O. Box 85500, 3508 GA Utrecht, The Netherlands; 2 Julius Center for Health Sciences and Primary Care, University Medical Center Utrecht, P.O. Box 85500, 3508 GA Utrecht, The Netherlands; 3 Department of Rheumatology, Tergooi Hospital, P.O. Box 10016, 1201 DA Hilversum, The Netherlands; 4 Department of Rheumatology, St. Jansdal Hospital, P.O. Box 138, 3840 AC Harderwijk, The Netherlands

## Abstract

**Introduction:**

The aim of this study was to investigate whether serum biomarker levels of C2C, C1,2C, CS846, and CPII can predict the long-term course of disease activity and radiographic progression early in the disease course of rheumatoid arthritis (RA).

**Methods:**

In patients in the CAMERA trial, levels of biomarkers were evaluated at baseline and after 1 year of treatment. Relations of (changes in) biomarker values with the mean yearly radiographic progression rate and mean disease activity over a 5-year period were evaluated by using regression analysis. The added predictive value of biomarkers over established predictors for long-term outcome was analyzed by multiple linear regression analysis.

**Results:**

Of 133 patients, serum samples were available at baseline and after 1 year of treatment. In the regression analysis C1,2C at baseline, the change in C2C, C1,2C, and the sum of the standardized changes in C2C + C1,2C scores were statistically significantly associated with the mean yearly radiographic progression rate; the change in CPII was associated with the mean disease activity over 5 years of treatment. In the multiple linear regression analysis, only the change in C1,2C was of added predictive value (*P *= 0.004) for radiographic progression. Explained variances of models for radiographic progression and disease activity were low (0.28 and 0.34, respectively), and the biomarkers only marginally improved the explained variance.

**Conclusions:**

The change in C1,2C in the first year after onset of RA has a small added predictive value for disease severity over a 5-year period, but the predictive value of this biomarker combined with current predictive factors is too small to be of use for individual patients.

## Introduction

Biomarkers are molecules or fragments that are released into biologic fluids during the process of tissue turnover and, for rheumatoid arthritis (RA), are considered to be indicative of degradation or synthesis of cartilage, bone, and synovial tissue [1]. Several serum biomarkers are on the market, including those provided by IBEX (Montreal, Quebec, Canada); C2C, C1,2C, CS846, and CPII [2-5]. These biomarkers might be good candidates because they directly reflect the bone and cartilage turnover rate in the (affected) joints of patients with RA. The two markers for collagen degradation originate from type II collagen (C2C) and from type I as well as type II collagen (C1,2C), reflecting cartilage and bone degradation. The marker for turnover originated from proteoglycan aggrecan (CS846) and the marker for synthesis of type II procollagen (CPII).

Earlier research with these biomarkers showed no consistent results regarding the predictive value for the long-term outcomes in (early) RA. Only six publications described the relation of (one of) these biomarkers with (long-term) radiographic (Table 1) or clinical (Table 2) outcome in RA [6-11]. The relation between these biomarker values and radiographic progression is inconsistent; some studies show a higher value in cases of higher radiographic progression [7,9,11], whereas others show a lower value in cases of higher radiographic progression [8] or show no association at all [7-11]. The same holds true for the relation between these biomarker values and disease activity over time [9].

**Table 1:** Overview of the literature on the (significant) relation between biomarker and radiographic progression

Author	Population	**No**.	Biomarker	Classification	Results
Syversen *et al*.^10^	RA ≤4 yr	136	C2C (baseline serum)	SHS rapid >1 vs. slow <1	NS
				(radiographic progression per yr, progression change baseline to 5 or 10 yr)	
Mullan *et al*.^9^	RA	45	C2C (baseline,1, 3, 6, 9,12-mo serum)		C2C ↑ at 1, 3 mo
	PsA	17	C1,2C	*SHS rapid >1.5 vs. slow <1.5*	C1,2C ↑ at 1, 3 mo
	(mean 11 yr, DAS28 >3.2)		CPII	*(radiographic progression at 1 yr)*	NS
			ΔCOL (ΔC2C + ΔC1,2C + ΔCPII)		ΔCOL ↑ at 1, 3, 6, 9 mo
Verstappen *et al*.^11^	RA ≤1 yr	87	C2C (1, 2, 3, 4-yr serum)		C2C ↑
			C1,2C	*66*^*th *^*= SHS >7.4 vs. 33*^*rd *^*percentile = SHS <2.3*	C1,2C ↑
			CS846	*(radiographic progression over 4-yr span)*	CS846 ↑
			CPII		NS
Ishiguro *et al*.^7^	RA	63	C2C (knee SF)	Mild vs. moderate vs. severe RA	NS
	(mean 10 yr)		CS846	Mild vs. moderate RA	CS846 ↓
			CPII	Mild vs. moderate vs. severe RA	NS
				(Larsen score: 0, 1 = mild; 2, 3 = moderate; 4, 5 = severe)	
Mansson *et al*.^8^	RA ≤2 yr	18	CS846 (baseline serum)	Rapid vs. slow hip-joint radiographic progression	CS846 ↓
			CPII	(Larsen score: rapid = 46; slow = 4 at 2 yr)	NS

Number, number of patients investigated in the studies; DAS28, disease activity score based on 28 joints; mo, month; NS, not significant. PsA, psoriatic arthritis; RA, rheumatoid arthritis; SF, synovial fluid; SHS, SharpvanderHeijde score; yr, year.

**Table 2:** Overview of the literature on the (significant) relation between biomarker and the disease activity

Author	Population	**No**.	Biomarker	Classification	Results
Mullan *et al*.^9^	RA	45	C2C (baseline,1, 3, 6, 9,12-mo serum)	*DAS28 responders vs. nonresponders (at 3 mo)*	C2C ↓
	PsA	17	C1,2C	*(responder: ≥0.6 improvement and DAS28 ≤5.1, nonresponder: <0.6 improvement OR DAS28 > 5.1)*	NS
	(mean 11 yr, DAS28 > 3.2)		CPII		NS
			ΔCOL (ΔC2C + ΔC1, 2C + ΔCPII)		ΔCOL ↓
Mullan *et al*.^9^	RA	45	C2C (baseline,1, 3, 6, 9, 12-mo serum)		C2C ↓ at 1 mo
	PsA	17	C1,2C	*Remission vs. no remission*	C1,2C ↓ at 1 mo
	(mean, 11 yr,		CPII	*(remission = DAS28 <2.6 at 6 mo)*	NS
	DAS28 > 3.2)		ΔCOL (ΔC2C + ΔC1,2C + ΔCPII)		ΔCOL ↓ change 1 mo

DAS28, disease activity score based on 28 joints; mo, month; NS, not significant; number, number of patients investigated in the studies; PsA, psoriatic arthritis; RA, rheumatoid arthritis; SF, synovial fluid; yr, year.

Because of these conflicting results and the limited available literature on the association between these biomarkers and clinical and radiographic progression, the aim of this study was to investigate whether values of C2C, C1,2C, CS846, and CPII determined early in the disease can predict the long-term radiographic and/or clinical outcome in patients with early RA.

## Materials and methods

Patients included in this study were participants in the 2-year randomized open-label prospective multicenter treatment strategy trial (Computer Assisted Management in Early Rheumatoid Arthritis, CAMERA) [12]. In the CAMERA study, patients were randomly assigned to either an intensive tightly controlled MTX-based treatment strategy based on computer-guided monthly predefined response criteria or to a conventional MTX-based treatment strategy based on regular clinical practice with 3-monthly visits. All patients fulfilled the 1987 revised American College of Rheumatology (ACR) criteria for RA [13]. At study entry, all patients had a disease duration of less than 1 year and were DMARD and glucocorticoid naïve. The medical ethics committees of all participating hospitals approved the study, and all patients gave written informed consent before entering the trial.

From all available patients, serum samples were collected at baseline (before treatment) and 1 year after inclusion into the study. Serum samples were frozen as soon as possible after blood collection and stored at -20°C until analysis (analysis shortly after all 1-year samples were obtained). Because the trial was performed according to general clinical practice as much as possible, sample collection was not restricted to fasting conditions.

### Biomarker analyses

For this study, only samples that had not been thawed before were used. For all biomarkers, enzyme-linked immunosorbent assays (ELISAs) were performed according to manufacturer's instructions (IBEX Montreal, Quebec, Canada).

The C2C serum ELISA detects a cartilage-specific collagen type II collagenase cleavage neoepitope [2]. The C1,2C ELISA detects a collagenase generated collagen type I and II cleavage neoepitope [3]. The CS846 ELISA detects an epitope on chondroitin sulfate of newly formed large aggrecan molecules [4]. The CPII ELISA recognizes epitopes of the propeptide of collagen type II reflecting synthesis [5].

Values of all four biomarkers were log transformed to obtain normal distributions. Additionally, seven extreme data point outliers derived from C2C, CS846, and CPII (based on visual inspection) were excluded for analysis.

### (Long-term) outcome measurements

The long-term outcome of RA patients was determined by the radiographic joint progression and by the mean disease activity over a 5-year period of treatment. To assess radiographic joint progression, radiographs of hand and feet were made at baseline and every subsequent year. Radiographs were independently scored according to the SharpvanderHeijde score (SHS) [14] by two readers, blinded to clinical information. The mean yearly radiographic progression rate between baseline and 5 years was used as the outcome measure. For this rate, if 5-year radiographs were not available, the mean of the measurements between 4 and 6 years was used, or scores at 4 or 6 years, depending on the data available. Because scores were not normally distributed, the log-transformed progression rate (log rate +1) was used.

The mean disease activity over a 5-year period was determined by calculating a time-averaged value of the DAS28 [15] by using the area under the curve (AUC) from baseline until 5 years after treatment. If more than two yearly time points were missing, no time-averaged DAS28 could be calculated.

The early response has been shown to be a predictor for long-term outcome [16] and was therefore also taken into account in the analysis. The DAS28 at baseline and 6 months was used to calculate the early EULAR response. Patients were classified as good, moderate, or nonresponders based on their early (change in) disease activity. Good responders should have a DAS28 score ≤3.2 at 6 months and an improvement from baseline >1.2; nonresponders a DAS28 score >5.1 and an improvement between 0.6 and 1.2 or only an improvement of ≤0.6. Patients with moderate response had a response in between the good responders and the nonresponders.

### Statistical analyses

The change in biomarker values was calculated by subtracting the baseline biomarker value from the 1-year value for all biomarkers. Furthermore, sum scores of (changes in) markers representing synthesis (CS846 and CPII) and sum scores of (changes in) markers for degradation (C2C and C1,2C) were calculated. Finally, the ratio of (the sum scores of) synthesis and degradation markers were calculated. Because ranges of individual biomarker values differ, Z-scores (calculated by subtracting the average value from the individual value divided by the standard deviation) were used for the sum and ratio scores.

The relation between the individual (change, sum, and ratio of) biomarker values and long-term outcome (that is, mean yearly radiographic progression rate and time-averaged DAS28) was investigated by linear regression analysis, adjusting for the treatment strategy (that is, intensive tightly controlled or conventional MTX-based strategy).

Second, to investigate whether biomarker values were of additional value over already known baseline predictors (rheumatoid factor (RF) and joint damage or disease activity at baseline, respectively), multiple linear regression analysis was used, adjusting for treatment strategy. The sum and ratio scores were considered in the analysis only when the individual biomarkers had a significant association with the outcome in the initial analysis. In the final model also, the early (6-month) EULAR response was added, by means of two dummy variables (good and moderate response, with nonresponse as the reference category).

The statistical software SPSS 15.0 was used for the analyses. A *P *value < 0.05 was considered statistically significant.

## Results

Of 133 patients in the CAMERA trial, unthawed serum samples were available at baseline and at 1 year of treatment. Of these patients, 75 had been treated according to an intensive, tightly controlled MTX-based strategy, and 58 patients according to a conventional MTX-based strategy. For five patients, no mean yearly radiographic progression rate could be calculated because of missing scores. For 11 patients, no time-averaged DAS28 could be calculated because more than two DAS28 scores were missing. Baseline characteristics of patients with missing data were not statistically significantly different from those of patients with complete data. Clinical characteristics and biomarker data of the patients are shown in Table 3. Note that radiographic progression is limited (median (IQR) radiographic progression rate over 5 years is 1.0 (0.0 to 3.4); mean (SD) value, 2.7 (4.5) SHS units).

**Table 3:** Clinical and biomarker characteristics obtained at baseline and follow-up of all available patients

Characteristic		No. = 133
Female gender (%)		87 (65)
Age (years)		53 (14)
RF positivity (%)		87 (65)
Baseline DAS28		5.6 (1.0)
Baseline joint damage		0.0 (0.0-0.0)
EULAR good responders (%)		50 (38)
EULAR moderate responders (%)		58 (44)
EULAR no responders (%)		24 (18)
Time-averaged DAS28		3.0 (0.9)
Radiographic progression rate		1.0 (0.0-3.4)
C2C (ng/ml)	Baseline	90 (71-124)
	1 yr	86 (70-109)
C1,2C (ng/ml)	Baseline	359 (286-427)
	1 yr	349 (269-415)
CS846 (ng/ml)	Baseline	100 (68-155)
	1 yr	113 (69-183)
CPII (ng/ml)	Baseline	335 (207-551)
	1 yr	436 (220-613)

Mean (SD) is shown for age and (baseline and time-averaged) DAS28; median (IQR) is shown for all other (non-normally distributed) continuous variables. For all categoric variables, number (%) of patients is shown. The EULAR response was determined after 6 months of treatment; time-averaged DAS28 and radiographic progression rate were calculated over 5 years of treatment; all other variables were determined at baseline unless otherwise stated. RF, rheumatoid factor; DAS28, disease activity score based on 28 joints.

In the analyses correcting for treatment strategy, C1,2C at baseline, the change in C2C and in C1,2C, and (consequently) the sum of the standardized changes in C2C + C1,2C levels were statistically significantly related to the mean yearly radiographic progression rate (all *P *< 0.05; Table 4). Only the change in CPII levels was related to time-averaged DAS28 (*P *= 0.03; Table 4).

**Table 4:** Association between biomarker values and the long-term outcome measures

		Long-term outcome (5 years after treatment)					
		Yearly radiographic progression rate			Time-averaged DAS28		
**Biomarker**		**No**.	**B**	**95% CI**	**No**.	**B**	**95% CI**
C2C	Baseline	126	0.08	-0.28 to 0.44	120	0.11	-0.25 to 0.47
	1 yr	126	-0.23	-0.67 to 0.20	120	0.18	-0.25 to 0.61
	Change	126	**-0.59**	**-1.14 to -0.03**	120	0.04	-0.52 to 0.60
C1,2C	Baseline	126	**0.47**	**0.001 to 0.95**	120	0.10	-0.40 to 0.60
	1 yr	127	0.14	-0.36 to 0.65	121	0.22	-0.29 to 0.73
	Change	126	**-1.00**	**-1.80 to -0.20**	120	0.33	-0.52 to 1.18
CS846	Baseline	127	-0.06	-0.27 to 0.16	121	-0.08	-0.29 to 0.14
	1 yr	126	-0.07	-0.29 to 0.15	120	-0.03	-0.26 to 0.20
	Change	126	-0.01	-0.23 to 0.21	120	0.05	-0.18 to 0.28
CPII	Baseline	124	0.14	-0.07 to 0.35	118	-0.05	-0.26 to 0.17
	1 yr	125	0.13	-0.09 to 0.34	119	0.10	-0.12 to 0.31
	Change	122	-0.07	-0.34 to 0.20	122	**0.30**	**0.02 to 0.57**
ZC2C + ZC1,2C	Baseline	125	0.07	-0.02 to 0.17			
	1 yr	126	0.01	-0.11 to 0.08			
	Change	126	**-0.13**	**-0.22 to -0.04**			

Biomarkers with B (95% confidence interval (CI)) values, which are shown in **Bold **type have a *P *value < 0.05 and have been included in the multiple regression analyses. Biomarkers values were determined at baseline, at 1 year, and the change between 1 year and baseline. Next are the sum and ratio scores (based on Z-values), determined when individual biomarkers had a significant association with the outcome in the initial analysis.

DAS28, disease activity score based on 28 joints; *n*, number of patients investigated; 95% CI, 95% confidence interval.

In the multiple linear regression analyses, the change in C1,2C and the sum of the standardized changes in C2C + C1,2C levels were significantly related (*P *= 0.004 and *P *= 0.02, respectively) to mean yearly radiographic progression rate in addition to RF, baseline joint damage, and early (6-month) EULAR response. However, when including both changes in biomarkers values in the analysis, they were no longer statistically significant (*P *= 0.13 and *P *= 0.94, respectively). The change in C1,2C was chosen for the final model because this biomarker had the highest standardized beta, and the final model had the highest R^2 ^when compared with the sum of the standardized changes in C2C + C1,2C levels; furthermore, including only one biomarker instead of two is more efficient.

The R^2 ^of the final model increased from 0.23 without biomarker to 0.28 including the change score of C1,2C (Table 5). When early response was not included, results were comparable, and the R^2 ^of the model changed from 0.20 to 0.27 if C1,2C was added. The standardized beta values showed that the influence of the biomarkers on prediction of the mean yearly radiographic progression rate was much smaller than, for instance, the predictive influence of baseline joint damage (standardized beta = -0.24 vs. 0.44, respectively; Table 5).

**Table 5:** Added predictive value of biomarkers over already known predictors for mean yearly radiographic progression rate over 5 years of treatment

Item	B	95% CI	Standardized beta	*P*	**R**^**2**^
Intercept	0.54	0.12 to 0.96		0.013	
Treatment strategy	0.13	-0.16 to 0.43	0.08	0.375	0.000
RF positive	0.29	-0.02 to 0.60	0.15	0.063	0.029
Baseline joint damage	0.09	0.06 to 0.13	0.44	**0.000**	0.211
EULAR good response^a^	-0.36	-0.78 to 0.07	-0.20	0.100	
EULAR moderate response^a^	-0.13	-0.52 to 0.27	-0.07	0.534	0.229
C1,2C change from 1 yr to baseline	-1.11	-1.87 to -0.36	-0.24	0.**004**	0.283

^a^EULAR nonresponse was used as reference category. 95% CI, 95% confidence interval; RF, rheumatoid factor.

The change score of CPII was not statistically significantly related (*P *= 0.18) to time-averaged DAS28. The R^2 ^of the model increased marginally from 0.32 without biomarker to 0.34 including this biomarker (Table 6). When early response was not included in the model, the R^2 ^increased from 0.13 to 0.21 by adding the biomarker, but CPII was still not statistically significantly related to time-averaged DAS28. The standardized beta values also showed that the influence of the biomarkers was much smaller than those of RF, baseline disease activity, and early EULAR response (Table 6).

**Table 6:** Added predictive value of biomarkers over already known predictors for time-averaged disease activity (DAS28) over 5 years of treatment

Item	B	95% CI	Standardized beta	*P*	**R**^**2**^
Intercept	1.90	0.99 to 2.81		0.000	
Treatment strategy	-0.29	-0.62 to 0.03	-0.16	0.076	0.076
RF positive	0.35	0.02 to 0.68	0.18	**0.040**	0.098
Baseline disease activity	0.25	0.10 to 0.40	0.30	**0.001**	0.169
EULAR good response	-0.84	-1.30 to -0.37	-0.46	**0.001**	
EULAR moderate response	-0.19	-0.64 to 0.25	-0.11	0.393	0.322
CPII change 1 yr to baseline	0.18	-0.08 to 0.43	0.12	0.178	0.335

EULAR nonresponse was used as reference category. 95% CI, 95% confidence interval; RF, rheumatoid factor.

## Discussion

The results show that some of the biomarkers have a small predictive value for long-term outcome in early RA, but clearly less, compared with established predictors. Only the change in C1,2C, the sum of the standardized changes in C2C + C1,2C levels, and the change in CPII were of added value for the mean yearly radiographic progression rate and the time-averaged DAS28, respectively. However, the explained variance of the final prediction models was low and therefore not useful for clinical practice, and both biomarkers increased the explained variance only marginally (and not statistically significantly for CPII).

Possible explanations for not finding a relation with all biomarkers are multiple. Importantly, it should be considered that blood for serum was collected during the day, which will influence the biomarker levels [17]. With respect to changes in biomarkers, it might have been worthwhile to evaluate changes in biomarkers within a shorter time span (for example, 3 or 6 months from baseline). However, no biologic samples were available at these time points. Also of relevance are the small variances in outcome regarding the radiographic progression due to the low radiographic scores, despite the 5 years of follow-up. We compared other investigations of the four biomarkers (see Tables 1 and 2) with our own data; patients in the other studies had higher radiographic scores at baseline and had, on average, also higher disease durations (varying from 1 to 10 years RA). The available radiographic scores at baseline of the evaluated studies range from 6.8 to 60 for SHS (mean) and 2 to 7 for the Larsen score (median) compared with 0 SHS (median) in our study. Verstappen *et al*. [11] investigated the same biomarkers comparing fast (>7.3 SHS units/year) and slow progressors (<2.3 SHS units/year) and found significant differences in biomarkers values, except for CPII, in another cohort of patients with early RA. However, these slowest progressors (calculated over a 4-year period) in this previous study are comparable to the patients with the fastest progression (66^th ^tertile >2.4 SHS units/year) in our present study (calculated over a 5-year period). Important to consider is that, because of improved treatment (strategies), the progression rate now in the Western community will hardly exceed the progression rate of the present cohort. This progress in treatment effectiveness and tight control strategies titrating treatment to the disease course of an individual patient might counterbalance the predictive value of biomarkers in prediction of disease outcome. However, it should not be ignored that also in the previous studies with higher radiographic progression rates, the relation of these biomarkers with outcome was not straightforward (see Table 1).

In a *post hoc *analysis evaluating all sum and ratio scores of synthesis and degradation markers (instead of only the ones when the individual biomarker had a significant association), no significant associations were seen with both the mean yearly radiographic progression rate and the time-averaged DAS28 over a 5-year period of treatment; this also applied for the multiple linear regression analysis (data not shown). The possible influence of age and gender on the biomarker values was also investigated with multiple linear regression analyses; adding these variables to the models did not change the results (data not shown). Using logistic regression analysis comparing progressors versus nonprogressors in radiographic progression (any radiographic damage at 5 years) also did not change the results (data not shown). When patients were selected with a minimum radiographic progression rate of 1 point per year (SHS of 5 units at 5 years), the relation with the biomarkers did not improve (data not shown). In case progression in joint space narrowing and erosions were taken apart, because biomarkers primarily represent cartilage turnover, no significant relations with biomarkers were found (data not shown).

Although glucocorticosteroid (GC) use was prohibited during the 2-year trial period; after 2 years, GC use was free. Only a limited number of patients used GC (*n *= 13). Because GC may influence joint damage significantly [18], analyses were performed in the group of patients not using GC during the 5 years of treatment. In these *post hoc *analyses, no clear relations between radiographic joint damage and biomarkers were found.

The direction of the relation between the biomarkers and the mean yearly radiographic progression rate and time-averaged DAS28 was not anticipated. An increase in C1,2C during 1 year of treatment, which indicates more connective tissue degradation, led to lower mean yearly radiographic progression rate, whereas a higher time-averaged DAS28 was reached with an increase in cartilage collagen synthesis, as determined by an increase in CPII between baseline and 1 year of treatment. Conversely, *in vitro *data reveal that the neoepitope can increase when collagenase activity is blocked [19]. This is because collagenase can cleave the neoepitope that it generates [3]. In osteoarthritis (OA) serum, CPII increased with progression of OA (Poole *et al*., unpublished data), similar to that in the present study on RA. As in general, contrasting relations have been found (Tables 1 and 2 and this study), clearly the nature, origin, and metabolism of these (and other) biomarkers require further investigation [20].

Based on the present results, the investigated markers are not the first choice in predicting long-term outcome in individual patients with early RA. The available studies together with the present results suggest that the role of these markers in predicting long-term outcome is at most modest. They might, conversely, be of value for other joint diseases or in distinguishing RA from other arthritis conditions. Significant differences in these biomarkers were reported when comparing RA with psoriatic arthritis [6], OA [6,7], and controls [8]. When we investigated the baseline biomarker values of the early RA patients of the CAMERA trial with controls, also significant differences were seen (all *P *< 0.01; data not shown). For assessment of progression in treatment with anti-TNF, these biomarkers appeared of use [9].

Biomarkers in general might be of value in prediction of the long-term outcome of RA. CTX-II [21-25], CTX-I [22,24], MMP-3 [25,26], COMP [27], calprotectin [28], RANKL [29,30], and IL-6 [31] all had a relation with (long-term) radiographic progression and/or the disease-activity score. Of all these biomarkers, urinary CTX-II is at present the most frequently used and best-performing marker. Recently, a trial demonstrated urinary CTX-II and DAS28 almost equally effective when used to monitor disease activity and in treatment decisions aiming at remission of disease of RA [32]. Unfortunately, in our study, no urine samples were obtained.

## Conclusions

In conclusion, the change in C1,2C and CPII in the first year after onset have a small added predictive value for radiographic progression and disease activity, respectively, over a 5-year period, although the predictive value is too small to be useful in daily clinical practice.

## Abbreviations

ACR: American College of Rheumatology; AUC: area under the curve; CAMERA: Computer Assisted Management in Early Rheumatoid Arthritis; DAS28: disease activity score based on 28 joints; DMARD: disease modifying anti-rheumatic drug; MTX: methotrexate; OA: osteoarthritis; RA: rheumatoid arthritis; RF: rheumatoid factor; SHS: SharpvanderHeijde score.

## Competing interests

The authors declare that they have no competing interests.

## Authors' contributions

MB, SV, PW, JJ, JB, and FL contributed to the conception and design of the study. MB and PW contributed to the analysis of data. MB, SV, PW, JJ, JB, and FL contributed to the interpretation of data. NJ and MV provided study participants. Article drafts were written by MB and critically revised by all authors. The final version of the manuscript was approved by all authors.

## References

[B1] Poole AR Dieppe P Biological markers in rheumatoid arthritis Semin Arthritis Rheum 1994 23 17 31 10.1016/0049-0172(94)90081-7 7939727

[B2] Poole AR Ionescu M Fitzcharles MA Billinghurst RC The assessment of cartilage degradation in vivo: development of an immunoassay for the measurement in body fluids of type II collagen cleaved by collagenases J Immunol Methods 2004 294 145 153 10.1016/j.jim.2004.09.005 15637808

[B3] Billinghurst RC Dahlberg L Ionescu M Reiner A Bourne R Rorabeck C Mitchell P Hambor J Diekmann O Tschesche H Chen J Van Wart H Poole AR Enhanced cleavage of type II collagen by collagenases in osteoarthritic articular cartilage J Clin Invest 1997 99 1534 1545 10.1172/JCI119316 9119997 PMC507973

[B4] Rizkalla G Reiner A Bogoch E Poole AR Studies of the articular cartilage proteoglycan aggrecan in health and osteoarthritis: evidence for molecular heterogeneity and extensive molecular changes in disease J Clin Invest 1992 90 2268 2277 10.1172/JCI116113 1281828 PMC443378

[B5] Nelson F Dahlberg L Laverty S Reiner A Pidoux I Ionescu M Fraser GL Brooks E Tanzer M Rosenberg LC Dieppe P Robin Poole A Evidence for altered synthesis of type II collagen in patients with osteoarthritis J Clin Invest 1998 102 2115 2125 10.1172/JCI4853 9854047 PMC509166

[B6] Fraser A Fearon U Billinghurst RC Ionescu M Reece R Barwick T Emery P Poole AR Veale DJ Turnover of type II collagen and aggrecan in cartilage matrix at the onset of inflammatory arthritis in humans: relationship to mediators of systemic and local inflammation Arthritis Rheum 2003 48 3085 3095 10.1002/art.11331 14613270

[B7] Ishiguro N Ito T Oguchi T Kojima T Iwata H Ionescu M Poole AR Relationships of matrix metalloproteinases and their inhibitors to cartilage proteoglycan and collagen turnover and inflammation as revealed by analyses of synovial fluids from patients with rheumatoid arthritis Arthritis Rheum 2001 44 2503 2511 10.1002/1529-0131(200111)44:11<2503::AID-ART430>3.0.CO;2-P 11710706

[B8] Mansson B Carey D Alini M Ionescu M Rosenberg LC Poole AR Heinegard D Saxne T Cartilage and bone metabolism in rheumatoid arthritis: differences between rapid and slow progression of disease identified by serum markers of cartilage metabolism J Clin Invest 1995 95 1071 1077 10.1172/JCI117753 7533784 PMC441442

[B9] Mullan RH Matthews C Bresnihan B FitzGerald O King L Poole AR Fearon U Veale DJ Early changes in serum type II collagen biomarkers predict radiographic progression at one year in inflammatory arthritis patients after biologic therapy Arthritis Rheum 2007 56 2919 2928 10.1002/art.22843 17763421

[B10] Syversen SW Goll GL van der Heijde D Landewe R Gaarder PI Odegard S Haavardsholm EA Kvien TK Cartilage and bone biomarkers in rheumatoid arthritis: prediction of 10-year radiographic progression J Rheumatol 2009 36 266 272 10.3899/jrheum.080180 19132792

[B11] Verstappen SM Poole AR Ionescu M King LE Abrahamowicz M Hofman DM Bijlsma JW Lafeber FP Radiographic joint damage in rheumatoid arthritis is associated with differences in cartilage turnover and can be predicted by serum biomarkers: an evaluation from 1 to 4 years after diagnosis Arthritis Res Ther 2006 8 R31 10.1186/ar1882 16507130 PMC1526568

[B12] Verstappen SM Jacobs JW van der Veen MJ Heurkens AH Schenk Y ter Borg EJ Blaauw AA Bijlsma JW Intensive treatment with methotrexate in early rheumatoid arthritis: aiming for remission: Computer Assisted Management in Early Rheumatoid Arthritis (CAMERA, an open label trial) Ann Rheum Dis 2007 66 1443 1449 10.1136/ard.2007.071092 17519278 PMC2111604

[B13] Arnett FC Edworthy SM Bloch DA McShane DJ Fries JF Cooper NS Healey LA Kaplan SR Liang MH Luthra HS The American Rheumatism Association 1987 revised criteria for the classification of rheumatoid arthritis Arthritis Rheum 1988 31 315 324 10.1002/art.1780310302 3358796

[B14] van der Heijde DM van Riel PL Nuver-Zwart IH Gribnau FW van de Putte LB Effects of hydroxychloroquine and sulphasalazine on progression of joint damage in rheumatoid arthritis Lancet 1989 1 1036 1038 2565997 10.1016/s0140-6736(89)92442-2

[B15] Prevoo ML van't Hof MA Kuper HH van Leeuwen MA van de Putte LB van Riel PL Modified disease activity scores that include twenty-eight-joint counts: development and validation in a prospective longitudinal study of patients with rheumatoid arthritis Arthritis Rheum 1995 38 44 48 10.1002/art.1780380107 7818570

[B16] Bakker MF Jacobs JWG Bijlsma JWJ Lafeber FPJG Early good response to therapy in RA patients predicts a better long-term clinical course and less radiographic joint damage Arthritis Rheum 2008 58 S764 S765 10.1002/art.23263

[B17] Kong SY Stabler TV Criscione LG Elliott AL Jordan JM Kraus VB Diurnal variation of serum and urine biomarkers in patients with radiographic knee osteoarthritis Arthritis Rheum 2006 54 2496 2504 10.1002/art.21977 16868970

[B18] Scott DL Pugner K Kaarela K Doyle DV Woolf A Holmes J Hieke K The links between joint damage and disability in rheumatoid arthritis Rheumatology (Oxford) 2000 39 122 132 10.1093/rheumatology/39.2.122 10725061

[B19] Dahlberg L Billinghurst RC Manner P Nelson F Webb G Ionescu M Reiner A Tanzer M Zukor D Chen J van Wart HE Poole AR Selective enhancement of collagenase-mediated cleavage of resident type II collagen in cultured osteoarthritic cartilage and arrest with a synthetic inhibitor that spares collagenase 1 (matrix metalloproteinase 1) Arthritis Rheum 2000 43 673 682 10.1002/1529-0131(200003)43:3<673::AID-ANR25>3.0.CO;2-8 10728762

[B20] van Spil WE DeGroot J Lems WF Oostveen JC Lafeber FP Serum and urinary biochemical markers for knee and hip-osteoarthritis: a systematic review applying the consensus BIPED criteria Osteoarthritis Cartilage 2010 18 605 612 10.1016/j.joca.2010.01.012 20175979

[B21] Hashimoto J Garnero P van der Heijde D Miyasaka N Yamamoto K Kawai S Takeuchi T Yoshikawa H Nishimoto N A combination of biochemical markers of cartilage and bone turnover, radiographic damage and body mass index to predict the progression of joint destruction in patients with rheumatoid arthritis treated with disease-modifying anti-rheumatic drugs Mod Rheumatol 2009 19 273 282 10.1007/s10165-009-0170-4 19452245

[B22] Landewe RB Geusens P van der Heijde DM Boers M van der Linden SJ Garnero P Arthritis instantaneously causes collagen type I and type II degradation in patients with early rheumatoid arthritis: a longitudinal analysis Ann Rheum Dis 2006 65 40 44 10.1136/ard.2004.035196 16126801 PMC1797999

[B23] Marotte H Gineyts E Miossec P Delmas PD Effects of infliximab therapy on biological markers of synovium activity and cartilage breakdown in patients with rheumatoid arthritis Ann Rheum Dis 2009 68 1197 1200 10.1136/ard.2008.096057 18713784

[B24] Syversen SW Haavardsholm EA Boyesen P Goll GL Okkenhaug C Gaarder PI van der Heijde D Kvien TK Biomarkers in early rheumatoid arthritis: longitudinal associations with inflammation and joint destruction measured by magnetic resonance imaging and conventional radiographs Ann Rheum Dis 2010 69 845 850 10.1136/ard.2009.122325 20233753

[B25] Young-Min S Cawston T Marshall N Coady D Christgau S Saxne T Robins S Griffiths I Biomarkers predict radiographic progression in early rheumatoid arthritis and perform well compared with traditional markers Arthritis Rheum 2007 56 3236 3247 10.1002/art.22923 17907159

[B26] Visvanathan S Marini JC Smolen JS St Clair EW Pritchard C Shergy W Pendley C Baker D Bala M Gathany T Han J Wagner C Changes in biomarkers of inflammation and bone turnover and associations with clinical efficacy following infliximab plus methotrexate therapy in patients with early rheumatoid arthritis J Rheumatol 2007 34 1465 1474 17552048

[B27] Lindqvist E Eberhardt K Bendtzen K Heinegard D Saxne T Prognostic laboratory markers of joint damage in rheumatoid arthritis Ann Rheum Dis 2005 64 196 201 10.1136/ard.2003.019992 15458956 PMC1755350

[B28] Hammer HB Odegard S Syversen SW Landewe R van der Heijde D Uhlig T Mowinckel P Kvien TK Calprotectin (a major S100 leukocyte protein) predicts 10-year radiographic progression in patients with rheumatoid arthritis Ann Rheum Dis 2010 69 150 154 10.1136/ard.2008.103739 19095696

[B29] Gonzalez-Alvaro I Ortiz AM Tomero EG Balsa A Orte J Laffon A Garcia-Vicuna R Baseline serum RANKL levels may serve to predict remission in rheumatoid arthritis patients treated with TNF antagonists Ann Rheum Dis 2007 66 1675 1678 10.1136/ard.2007.071910 17666448 PMC2095335

[B30] van Tuyl LH Voskuyl AE Boers M Geusens P Landewe RB Dijkmans BA Lems WF Baseline RANKL:OPG ratio and markers of bone and cartilage degradation predict annual radiological progression over 11 years in rheumatoid arthritis Ann Rheum Dis 2010 69 1623 1628 10.1136/ard.2009.121764 20525836

[B31] Knudsen LS Klarlund M Skjodt H Jensen T Ostergaard M Jensen KE Hansen MS Hetland ML Nielsen HJ Johansen JS Biomarkers of inflammation in patients with unclassified polyarthritis and early rheumatoid arthritis: relationship to disease activity and radiographic outcome J Rheumatol 2008 35 1277 1287 18597410

[B32] van Tuyl LH Lems WF Voskuyl AE Kerstens PJ Garnero P Dijkmans BA Boers M Tight control and intensified COBRA combination therapy in early rheumatoid arthritis: 90% remission in a pilot trial Ann Rheum Dis 2008 67 1574 1577 18625629 10.1136/ard.2008.090712

